# Robotic Cardiac Surgery in Colombia: Overcoming the Challenges of a Middle-income Setting

**DOI:** 10.21470/1678-9741-2020-0064

**Published:** 2020

**Authors:** Darío Andrade, Eric E. Vinck, Juan F. Parra, Husam H. Balkhy, Federico Núñez

**Affiliations:** 1 Department of Cardiac Surgery, Fundacion Clinica Shaio, Bogotá, Colombia.; 2 Department of Cardiac Surgery, University of Chicago Medicine, Chicago, Illinois, United States.

**Keywords:** Robotic Surgical Procedures, Robotics, Cardiac Surgical Procedures, Colombia, Health Resources, Poverty

## Abstract

In developing countries, limited resources and low health budgets result in slow developments in the field of cardiac surgery. As a consequence, advances in surgery become a challenging process. In Colombia, most institutions do not have the capacity or infrastructure for minimally invasive and video-assisted cardiac surgery, let alone robotic assisted cardiac surgery (RACS). Despite the challenges, efforts to overcome these hurdles are critical for the future of cardiac surgery in low-income settings. Here we describe the first cases of robotic cardiac surgeries performed in Colombia.

**Table:** 

Abbreviations, acronyms & symbols	
ASD	= Atrial septal defects
CPB	= Cardiopulmonary bypass
FDA	= Food and Drug Administration
LVEF	= Left ventricular ejection fraction
ICU	= Intensive care unit
MICS	= Minimally invasive cardiac surgery
NYHA	= New York Heart Association
RACS	= Robotic-assisted cardiac surgery
VATS	= Video-assisted thoracoscopic surgery

## INTRODUCTION

Following the world’s first robotic cardiac surgery (RACS) in 1998, many countries began developing RACS departments, primarily middle and high-income countries. By 2002, the da Vinci console (Intuitive, Sunnyvale, California) received FDA approval for cardiac procedures ^[1,2]^. In Latin America, robotic heart surgery followed closely behind; in 2010 Brazil became the first Latin American country to perform robotic cardiac surgery. Although Colombia began performing minimally invasive (MICS) and video-assisted cardiac procedures since the mid 1990s, they were not routine until 2010. With regard to advances in minimally invasive cardiothoracic surgery, the country has come a long way from long traditional incisions to video-assisted and minimally invasive cardiac and thoracic surgeries, robotic thoracic surgery in 2012, and uniportal video-assisted thoracoscopic surgery (VATS) in 2014^[1-3]^. Although robotic cardiac procedures offer many advantages to patients, this approach is still finding its place, even in high-income countries. In low-income settings, limited resources and low health budgets result in slow developments in the field of cardiac surgery. As a consequence, advances in surgery become a challenging process^[1]^. In Colombia, most institutions do not have the capacity or infrastructure for MICS/ video-assisted cardiac surgery, let alone RACS. Here we describe the first cases of robotic cardiac surgeries performed in the developing country of Colombia.

### Patient series presentation

**Figs. 1A, B and C:**
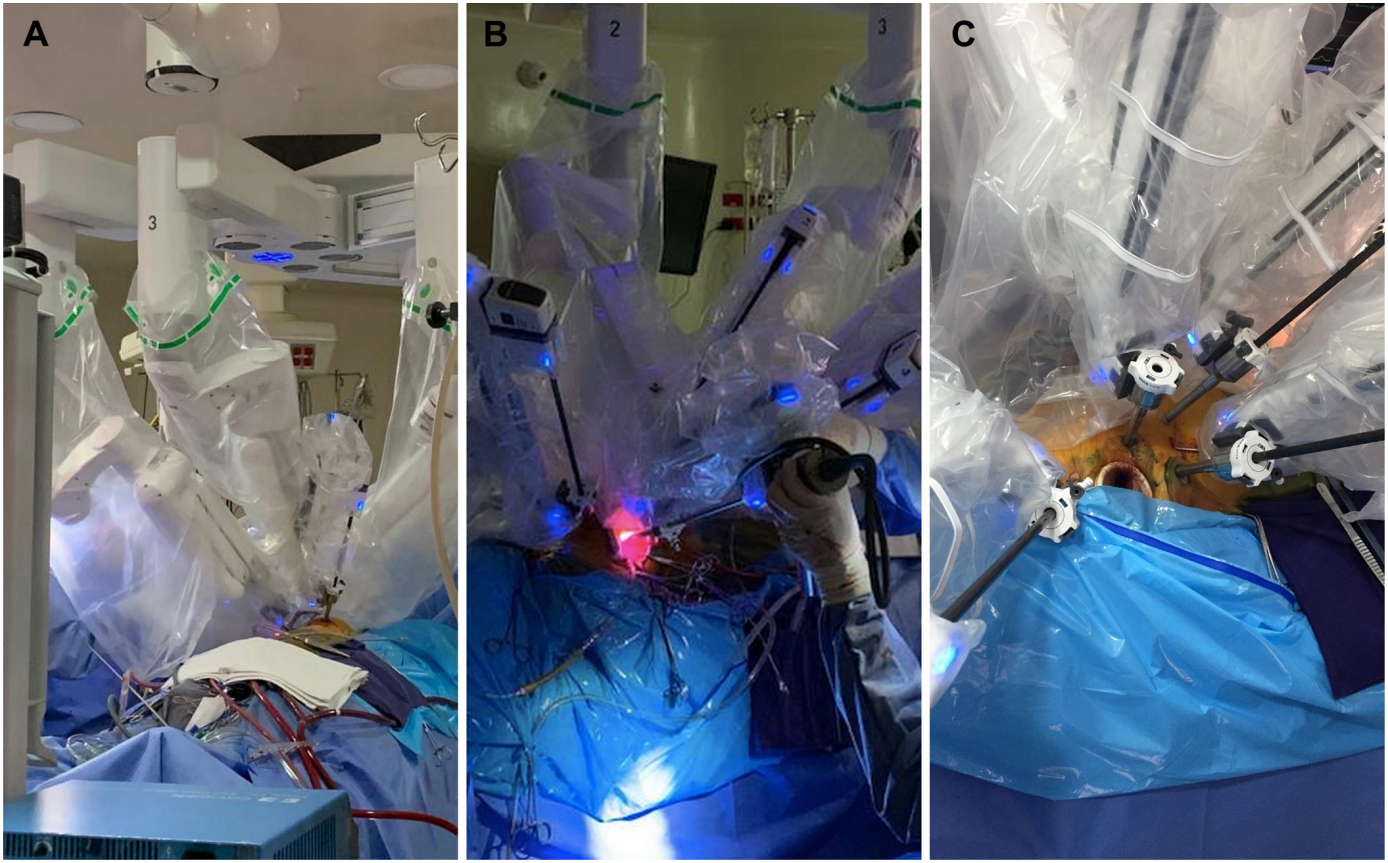
Robotic cardiac surgery arm docking and placement.

From September 2017 through 2018, six patients were taken to RACS at a single Cardiovascular and ECMO referral center in the Capital of Colombia, using a da Vinci Xi console (Intuitive, Sunnyvale, California) (Figure 1A to C). Of the 6 patients, three were female and three were male, three had severe symptomatic P2 mitral valve prolapse and the other three had secundum type symptomatic atrial septal defects (ASD). Because our RACS program was just starting, inclusion criteria for the best candidates were noncomplex, low comorbidity cases, thus patients in otherwise good general health requiring relatively simple surgeries. The patients taken to robotic mitral valve repairs had severe, symptomatic P2 prolapse, needing simple mitral valve repairs. All patients underwent preoperative chest tomography to rule out any vascular abnormalities, however, neither general thoracic anatomy nor chamber dimensions were determining inclusion or exclusion criteria for patient candidacy.

**Fig. 2:**
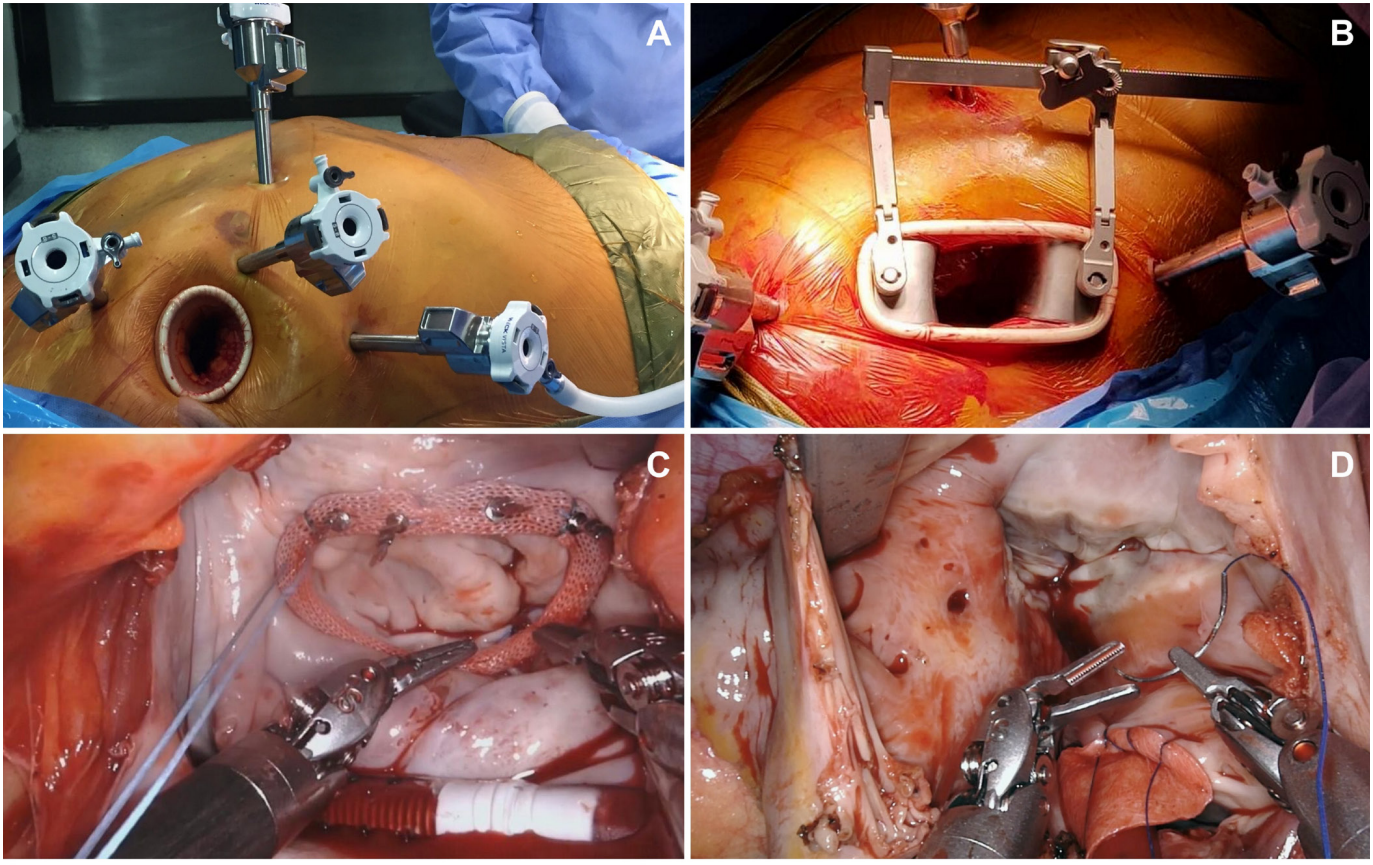
Lateral endoscopic approach using robotic (LEAR) surgical technique. (A) Total thoracoscopic 5-port technique. (B) 4-port approach with direct aortic (Chitwood) clamping. (C) Robotic view of mitral valve repair. (D) Robotic view of ASD repair.

The average age was 55.6±2.3 years and the primary comorbidity was systemic arterial hypertension. The patients had a NYHA functional class of I-II, preoperative LVEF averaged 63.2% and mean preoperative creatinine was 0.86 mg/dl. Cardiopulmonary bypass (CPB) was achieved through the right femoral artery and vein cannulation, as well as right internal jugular vein access. The first three procedures (two mitral valve repairs and one ASD repair) were performed by complete total thoracoscopy using an aortic endoclamp through a five-port approach (Level 4) (Figure 2A). For the other three procedures, direct aortic cross-clamping (Chitwood clamp) through a four-port approach (Level 2) was used (Figure 2B). The two techniques were 4-5 portal, with docking at the 2^nd^, 4^th^, and 6^th^ intercostal spaces on the anterior axillary line, an anterior chest port incision, along with a 3-4 cm incision at the 4^th^ IC space between anterior axillary line and mid-clavicular line (lateral endoscopic approach using robotics - LEAR surgery) (Figure 2A and 2B). Mean CPB time was 248.66 (173-295) minutes for mitral valve repairs (Figure 2C) and 175.66 (131-246) minutes for ASD repairs (Figure 2D). Aortic cross-clamp time was 171.66 (114201) minutes for mitral valve repairs and 81.66 (65-110) minutes for ASD repairs. Five patients had immediate extubation at the end of the surgery and the other patient was extubated six hours following surgery; no significant postoperative bleeding was reported. One ASD patient developed atrial fibrillation following surgery, medically treated. Mean postoperative ICU stay was two days and patients were discharged on postoperative day 3. All six patients were discharged on oral analgesics and returned to their daily activities within 8 days following RACS. In one year postoperatively, no complications were documented, no patients were reintervened; there were no residual mitral insufficiencies or mortality. Two other ASD repairs using robotic surgery have recently been successfully performed in a total of 8 cases; the patients are being followed up.

## COMMENTS

In Colombia, the dilemma of how to balance the care of low-income cardiac patients while simultaneously advancing technology in cardiac surgery is a constant challenge and raises the question of whether having robotic consoles in developing countries is cost-effective. This series represents the first experience in Colombia with robotic heart surgery paralleling reports in other countries with regard to effectiveness and safety ^[4]^. In comparison to traditional surgery, using a robotic console in Colombia costs an extra USD 2,044.67 for ASD repairs, USD 2,200.00 for mitral valve repairs, in addition to USD 6,000.00 when a total thoracoscopic approach using an aortic endoclamp is used. In the country, traditional (sternotomy) mitral valve repairs cost roughly USD 10,000.00; patients spend an average of five days in the hospital, three days in the ICU and two days on the floor for an additional USD 2,800,00, and return to daily activities in 3-4 weeks after surgery. RACS mitral valve repairs cost USD 19,600.00 using a total thoracoscopic technique and USD 13,667.00 per surgery using a hybrid approach. Despite the added costs of RACS in Colombia, our patients spent one day less in the coronary unit, one day less in the hospitalization ward, totaling USD 1,600.00 (43% less than traditional surgery) and returned to daily activities at 1-2 weeks after surgery (twice as fast as traditional surgery). RACS therefore translates into reduced overall hospital costs and work leave in comparison to traditional approaches, which is ideal in developing countries.

In order to provide patients with the most advanced cardiac surgical approaches while still maintaining an affordable cost-effective treatment, we applied a hybrid technique using direct aortic clamping with a Chitwood clamp, reducing the costs of RACS in Colombia by USD 6,000.00. Therefore, a hybrid approach, at least for now, may be the more appropriate next step in a low-income setting^[5,6]^. Despite these efforts to make RACS affordable, most insurance companies are unwilling to pay for the extra costs of robotic surgery through an inaccurate perception of hospital spending. To add to this scenario, national health coverage does not always authorize robotic surgeries limiting this option for many patients.

At the 5 institutions in Colombia with robotic consoles, robotic surgery is carried out mostly by various specialties, including gynecology, urology, head and neck, hepatobiliary and general thoracic surgery. With regard to the high operational costs of RACS as a new technology in the country, the insurance companies are less willing to pay for this method, however, because robotic surgery in Colombia, as in other countries, is a multispecialty technology, the extra costs of the console itself are generally covered by these other specialties. Therefore, at smaller centers with few surgical specialties, cardiac robotic surgery alone is not sustainable.

Although the steps forward in surgical technology are important, because of limited resources, the proper distribution of health budgets and coverage becomes fundamental to ensure that an already vulnerable population has the affordable cardiac care they need. Notwithstanding, government policies and competing public health priorities often limit these areas of growth^[4-6]^. Robotic cardiac surgery in developing countries, however, offers important advantages. First, it provides surgeons with a significantly more ergonomic and comfortable procedure with enhanced view, in turn, reducing human error and giving patients an ideal surgical and postoperative experience. Second, limiting the most minimally invasive and technologically advanced techniques to high-income patients only and providing a low-income population with cheaper more traumatic incisions is a socioeconomic problem that needs to change. Pursuing the most optimal approaches for all patients, regardless of their health coverage, impulses and guarantees a more universal approach to the highest standards and quality of care. Finally, having robotic cardiac surgery in a developing country such as Colombia triggers technological growth and generates opportunities for other areas of surgical advancements.

Throughout the years, health care reimbursement has evolved from a fee-for-service to a more fee-for-performance model with regard to disease specific coverage and payments. This drives institutions to develop policies that reinforce value-driven patient care^[7]^. In developing countries, health care spending, if not carefully managed, can quickly result in unbalanced priorities and distribution of resources ultimately affecting patients. As the pursuit of optimal and universal health care continues, the next step with regard to optimizing access to quality cardiac surgical care pushes more towards bundled payment models^[7]^. At the moment, robotic surgery in Colombia is still in its infancy, with only 5 centers in the country having robotic consoles, and only one center performing robotic cardiac surgery^[1-3,5]^. Throughout the years, cardiothoracic surgery in Colombia has grown following American footsteps and techniques. From the country’s first heart transplantation using techniques from Stanford, to lung transplant surgery in Bogotá following Duke surgical approaches, and now robotic cardiac surgery learned from the University of Chicago Medicine; just to name a few^[1,5]^. In essence, Colombia owes a great part of its cardiothoracic evolution to North American pioneers and centers. To ensure the continued growth of RACS in the country, attention needs to be kept first and foremost on the needs of “the patient” and recognize the importance of international/ visiting RACS teams.

## CONCLUSION

Although efforts are constantly being made to improve patient care with newer technology, being a developing country has well-recognized challenges. Despite these difficulties, efforts to overcome the financial hurdles are critical for the future of cardiac surgery in low-income settings. As cardiac surgery in Colombia keeps on advancing with the resources available following American and European standards, robotic cardiac surgery will keep on searching for its place in both high and lowincome countries.

**Table:** 

**Authors' roles & responsibilities**	
DA	Substantial contributions to the conception or design of the work; or the acquisition, analysis or interpretation of data for the work; final approval of the version to be published
EEV	Substantial contributions to the conception or design of the work; or the acquisition, analysis or interpretation of data for the work; drafting the work or revising it critically for important intellectual content; final approval of the version to be published
JFP	Drafting the work or revising it critically for important intellectual content; final approval of the version to be published
HHB	Substantial contributions to the conception or design of the work; or the acquisition, analysis or interpretation of data for the work; final approval of the version to be published
FN	Final approval of the version to be published
